# Consensus Interferon Plus Ribavirin for Hepatitis C Genotype 3 Patients Previously Treated With Pegylated Interferon Plus Ribavirin

**DOI:** 10.5812/hepatmon.14146

**Published:** 2013-12-14

**Authors:** Zaigham Abbas, Ghiasun Nabi Tayyab, Mustafa Qureshi, Mohammad Sadik Memon, Amna Subhan, Tanzila Shakir, Wasim Jafri, Saeed Hamid

**Affiliations:** 1 Department of Medicine, The Aga Khan University Hospital, Karachi, Pakistan; 2 Postgraduate Medical Institute, Lahore, Pakistan; 3 Department of Medicine, Isra University Hospital, Hyderabad, India

**Keywords:** Hepatitis C, Genotype, Ribavirin, Treatment

## Abstract

**Background:**

Not enough data are available about the effectiveness of consensus interferon (CIFN) among HCV genotype 3 patients who failed to respond to pegylated interferon and ribavirin.

**Objectives:**

We aimed to assess the efficacy and safety of CIFN and ribavirin in non-responders and relapsers to pegylated interferon with ribavirin therapy.

**Patients and Methods:**

This open-label investigator-initiated study included 44 patients who received CIFN 15 µg /day plus ribavirin 800-1200 mg daily. In patients with an early virological response (EVR), the dose of CIFN was reduced to 15 µg thrice a week for further 36 weeks. Patients with delayed virological response continued to receive daily CIFN plus ribavirin to complete 48 weeks. The patients were considered “non-responders” if there were less than 2 log reduction in HCV RNA at 12 weeks and detectable HCV RNA at 24 weeks.

**Results:**

Twenty-four patients (55%) were non-responders and 20 patients were relapsers to the previous treatment with pegylated interferon plus ribavirin (mean age 43.6 ± 9.4 years, males 25 (57%)). Nine patients were clinically cirrhotic (Child A). End of treatment virological response was achieved in 19 (43.1%) patients and sustained virological response (SVR) occurred in 12 (27.3%). Out of these 12 patients, eight were non-responders and four were relapsers to the previous treatment. Advanced fibrosis or clinical cirrhosis was associated with low SVR. Adverse events were fever, myalgia, anorexia, depression, and weight loss. Two patients received granulocyte colony stimulating factor for transient neutropenia. Seven patients were given erythropoietin to improve hemoglobin, and six were treated for mild depression. Two patients developed portosystemic encephalopathy.

**Conclusions:**

More than one-quarter of treatment-experienced patients with HCV genotype 3 achieved SVR after re-treatment with consensus interferon plus ribavirin.

## 1. Background

Hepatitis C virus (HCV) infection is the second most common chronic viral infection affecting 170 million people worldwide (1). It is responsible for 25-30% cases of cirrhosis globally. The resultant cirrhosis is associated with increasing risk of hepatic decompensation and hepatocellular carcinoma (HCC) (2). Sustained virological response (SVR) after antiviral therapy may halt the progression of fibrosis with lower risk of developing HCC and improve survival (3). However, the SVR rates depend upon many host- and virus-related factors, including age, gender, obesity, IL-28B genotype, stage of liver fibrosis, HCV genotype, and baseline viral load (2, 4, 5). Treatment with pegylated interferon (Peg-IFN) and ribavirin (RBV) is considered as the standard treatment for hepatitis C associated with SVR in 40-50% and up to 80% of HCV genotype 1 and 2/3 (naïve) patients, respectively (6-8). Additionally, re-treatment with Peg-IFN and RBV can also lead to SVR in 6-15% of non-responders and 32-50% of relapsers to previous treatment with standard interferon with or without RBV3, (3, 9, 10). 

Chronic hepatitis C (CHC) patients who are non-responders or relapsers to Peg-IFN and RBV are the most challenging population that hepatologists face with, and the optimal approach for treatment of these patients would be the use of direct-acting antiviral agents (DAA) with or without Peg-IFN and RBV. Several alternative approaches were attempted in pre-DAA era such as re-treatment with alternative brand, prolonged treatment with Peg-IFN, maintenance therapy, or use of higher doses of Peg-IFN with or without RBV (10-12). However, the results were not promising in the majority of such approaches (2, 13). Another drug modality considered in some studies was consensus interferon (CIFN) with or without RBV (9, 12). 

CIFN is a synthetic, recombinant type-I interferon with 166 amino acids and molecular weight of 19,500 dalton engineered by creation of a consensus sequence involving the most common amino acids found in naturally occurring alpha interferon subtypes (14). In in-vitro cell lines, CIFN has shown 10 fold greater antiviral efficacy than naturally occurring by IFN alpha, and may have finer efficacy in difficult-to-treat CHC patients (3, 15, 16). Due to differences in dosing, heterogeneity in study populations, and lack of comparative data with Peg-IFN plus RBV, CIFN is not considered as the first-line agent for treatment of HCV, although it may have a potential role in the management of CHC patients who failed to respond to previous interferon-based therapy (15, 17). Studies evaluating the efficacy of CIFN in standard IFN therapy failure with or without RBV have shown SVR of 5-33% and 28-58% with CIFN monotherapy among non-responders and relapsers, respectively (18-21), while in RBV-added regimen the SVR was further improved to 22-39% in non-responders but remained at 26-47% in relapsers (22-26). Most of the patients in these studies were infected with HCV genotype 1.

In Pakistan, HCV infection has been reported to affect approximately 10 million people and is the most common cause of cirrhosis and HCC (27). The HCV genotype type 3 is the most prevalent genotype affecting 67-87% of cases (28). HCV genotype 3 was considered as easier to treat; however, such data were extrapolated from subgroup analysis in larger trials, which were mostly conducted on a Caucasian population with genotype 1. In our clinical practice, we often encounter the issue of treatment failure in patients with HCV genotype 3 even after treatment with Peg-IFN and RBV. In addition, data collected from the use of CIFN plus RBV therapy in patients with CHC due to genotype 3, and from relapsers to Peg-IFN + RBV therapy are scanty. Hence, there is a need to evaluate the efficacy and tolerability of CIFN and RBV combination therapy in CHC genotype 3 patients who relapsed or failed to respond to previous treatment with Peg-IFN and RBV.

## 2. Objectives

The objectives of this study were to assess the efficacy and safety of CIFN and RBV in patients with chronic hepatitis C genotype 3 who were non-responders or relapsers to previous therapy with Peg-IFNα 2a or 2b and RBV.

## 3. Patients and Methods

### 3.1. Study Design

This was a phase 4 open-label investigator-initiated clinical trial to investigate the efficacy and safety of consensus interferon (CIFN) and ribavirin (RBV) therapy in the treatment-experienced patients who were non-responders or relapsers to Peg-IFN and RBV. The study was conducted at three tertiary care centers in the cities of Karachi, Lahore, and Hyderabad, Pakistan. 

### 3.2. Inclusion and Exclusion Criteria

The study population included consecutive patient’s ≥ 18 years of age with chronic hepatitis C (CHC) infection due to genotype 3 who were non-responders or relapsers to previous therapy with Peg-IFNα 2a or 2b plus ribavirin. The diagnosis of CHC was based on detectable anti-HCV antibody (by ELISA-IV or MEIA method) and serum HCV RNA by PCR (COBAS Amplicor, HCV qualitative assay) with normal or elevated ALT. The patients were eligible for the study in the absence of a prior episode of hepatic decompensation given that they had normal liver function evident by serum bilirubin < 2 mg/dL, serum albumin ≥ 3. 5 g/dL, and platelet count ≥ 75 × 103 /mcL. Patients with upper gastrointestinal bleeding due to esophageal varices eradicated after serial esophageal variceal band ligations with or without beta-blockers, were also accepted for the study.

Patients were excluded from the study who had associated HBV, HDV, or HIV infection, HCV related decompensated cirrhosis defined as ascites, portosystemic encephalopathy, hepatorenal syndrome, HCC, and recurrent variceal bleeding which required premature discontinuation or dose reduction of Peg-IFN during previous treatment due to safety or tolerability issues, major psychiatric illness, hemoglobin < 10 g/dL for females and 12gr/dL for males, WBC counts < 2.5 × 10^3^ /mcL or neutrophil count < 1.5 × 10^3^ /mcL , platelets count < 75 × 10^3^ /mcL, serum creatinine > 1.5 mg/dL, concomitant metabolic or autoimmune liver disease, post liver transplant patients, pregnant and lactating females, uncontrolled seizures, severe heart disease, or other absolute contraindications for the treatment.

### 3.3. Study Procedures

All consecutive patients who have visited the study centers for treatment of hepatitis C were evaluated. Those who were found eligible and agree to participate in the study were enrolled after informed consent. Baseline medical history was recorded, physical examination was done, and blood was drawn for baseline laboratory tests including complete blood count (CBC), prothrombin time (PT), liver function tests (LFTs), serum creatinine, serum albumin, fasting blood sugar (FBS), and thyroid stimulating hormone (TSH). All nucleic acid tests were performed in the Clinical Laboratory of Karachi center using standard techniques. HCV genotyping was done by HCV-PCR reverse hybridization (INNOLIPA) technique, and plasma HCV RNA levels by Real Time quantitative assays. Liver biopsy was recommended for all patients, however, only 22 patients underwent for liver biopsy. Liver biopsies were interpreted by a single experienced histopathologist based in Karachi, using METAVIR scoring system (29). All information was collected using a preformed data collection form.

### 3.4. Study Medication and Protocol

**Figure 1.**
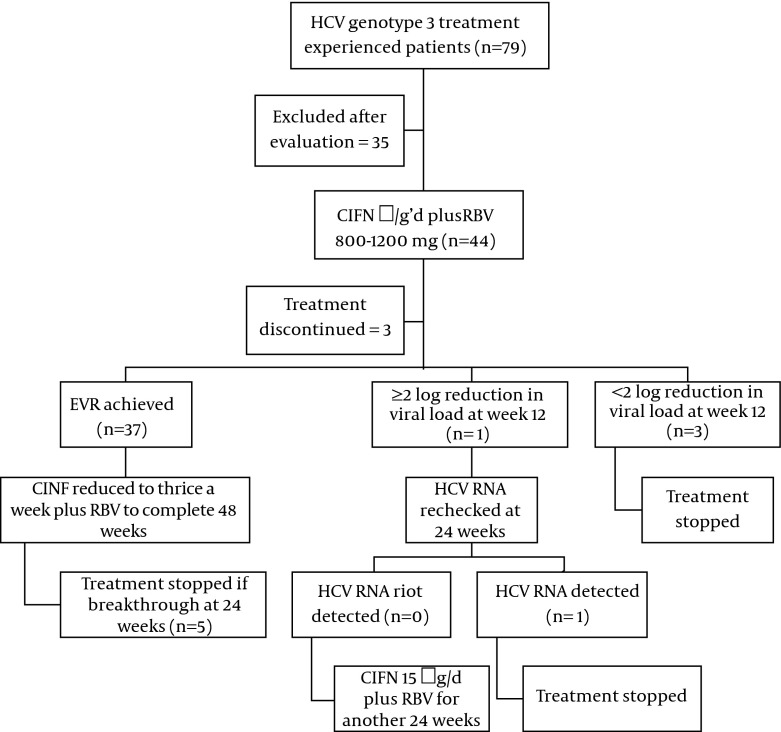
The Study Algorithm

Consecutive, eligible patients who agreed to participate in the study received CIFN 15 µg/day (INFERGEN; Three Rivers Pharmaceuticals, LLC, USA) subcutaneously along with RBV. RBV was given as 800 mg/day for body weight less than 70 kg and 1200 mg/day for body weight ≥ 70 kg in 2-3 divided doses. If the patients showed undetectable plasma HCV at week 12 (early virological response or EVR), the dose of CIFN was reduced to 15 µg thrice a week for further 36 weeks. However, the patients were considered “non-responders” and treatment was discontinued if there was a < 2 log10 reduction in HCV RNA from baseline at week 12. The patients with partial or delayed virological response (i.e. patients with detectable HCV RNA at week 12 but with ≥ 2 log10 reduction in HCV RNA from baseline) continued to receive CIFN 15 µg daily + RBV for an additional 12 weeks (i.e. till week 24). The treatment was discontinued when HCV RNA was detectable at week 24, and continued for responders by receiving CIFN 15 µg daily + RBV for further 24 weeks (Figure 1). Furthermore, end of treatment virological response (ETR) was assessed after the completion of 48-week therapy and patients were followed at week 72 to assess sustained virological response (SVR). 

### 3.5. Patient’s Monitoring and Follow-Up

Patients were assessed in outpatient clinics, initially twice weekly for one month and then every four weeks until the end of treatment. Once the treatment ended, patients were followed at weeks 12 and 24 post-treatment. Physical signs for hepatic decompensation, adverse effects of the antiviral therapy, complete blood count, and ALT were recorded at each visit. To detect thyroid dysfunction that might develop during treatment with CIFN, TSH was rechecked at week 12. Qualitative HCV PCR was checked at the weeks 12, 24, end of treatment, and 24 weeks afterwards. Moreover, to differentiate between non-responders and partial responders, plasma HCV RNA by Real Time quantitative assay was also checked at week 12 for those who demonstrated detectable qualitative HCV PCR. Treatment was terminated in case of clinical hepatic decompensation, or hemoglobin < 7.0 g/dL, platelets < 50 × 103 /mcL, and absolute neutrophil count (ANC) of < 0.5 × 103 /mcL. However, erythropoietin and/or granulocyte-colony stimulating factor (G-CSF) was given in situations where hemoglobin was < 8 g/dL and ANC < 0.75 × 109 /mcL. Moreover, the dose of CIFN and/or RBV were reduced in case of persistence Hb < 8 g/dL and absolute neutrophil count (ANC) < 0.75 × 103/mcL despite the addition of erythropoietin and ribavirin. Clinical decompensation is defined as development of ascites, hepatic hydrothorax, portosystemic encephalopathy (PSE), or variceal bleeding during treatment.

### 3.6. Outcome Measures 

Our primary outcome was SVR. Secondary outcomes included ETR, comparison between non-responders and responders to therapy, drug tolerability, and safety. 

### 3.7. Safety and Tolerability

All adverse events (AEs) and major adverse events were recorded for patients who received at least one dose of study medication. AEs were recorded until 30 days after the last dose of study medication. AEs were graded from 1 to 5 (1, mild; 2, moderate; 3, severe; 4, life-threatening or disabling; 5, death) based on the Common Terminology Criteria for Adverse Events v4.03 (30). An AE was considered as serious if it resulted in death, could be life-threatening, required hospitalization, or resulted in persistent or significant disability or incapacity.

### 3.8. Ethical Consideration

The study was conducted by maintaining compliance with the Helsinki Declaration, and was approved by the Ethical review committee of The Aga Khan University Hospital and collaborating centers (1643-MED-ERC-2010). Aims and objectives of the study, duration of treatment, required laboratory assessment, and risks and benefits associated with the study drugs were explained in detail. Patients were enrolled after informed consent. The baseline laboratory tests including liver biopsy were those tests that were routinely performed while treating such patients; hence these baseline laboratory tests were made at the patient’s own expense. However, the laboratory tests required during follow-ups and the study drugs were covered by study budget. Moreover, in case of serious adverse events including hepatic decompensation, cardiac toxicity, and bone marrow toxicity requiring hospitalization, or adjuvant treatment with erythropoietin or G-CSF, the cost was covered by study budget. The funding agency had no access to data and was not involved in data analysis or in writing manuscript. 

### 3.9. Sample Size and Statistical Analysis

The sample size was calculated by assuming an overall SVR rate of 21% (27) after treatment with CIFN and RBV, with 95% confidence level and a bound on error of ± 8%; an estimated sample size of 100 patients was required to achieve a 90 % power at a 5% significance level. However, due to funding issues, we decided to conduct a pilot study by taking 40% of required sample size (i.e. 40 patients).

Data were entered and analyzed by using SPSS for windows version 17 (SPSS Inc., Chicago, Illinois, USA). Results for quantitative variables were presented as mean ± standard deviation, median, or range after checking the normality and frequencies (percentages) for qualitative variables. Intention to treat analysis (ITT) and per-protocol analysis (PP) was performed to estimate SVR in the study population. Comparisons were done using Chi-square test or Fisher exact test for categorical variables, or student t-test for continuous variables. While applying latter test, Levene’s test was used to assess equality of variances. A P value < 0.05 was taken as significant. All values were two-tailed. To evaluate potential predicting factors for SVR, univariate and multivariate logistic regression analyses were performed. A P value < 0.05 was taken as significant. 

## 4. Results

During the recruitment phase, 79 consecutive patients were considered for inclusion in the study; 35 patients were excluded : 7 patients for previous episodes of decompensation, 11 for low platelets, 8 for low hemoglobin, 2 for elevated serum creatinine, 3 for being HBsAg positive, 1 for heart disease, and 3 patients who refused to give consent. Forty-four patients fulfilled the eligibility criteria and were enrolled, among them 24 patients (55%) were non-responders and 20 (45%) were relapsers to the previous treatment with Peg-IFN plus RBV. The overall mean age was 43.7 ± 9.4 years and 25 patients (57%) were male. The median baseline HCV viral load was 7.20x105 IU/ml (range 6.47 × 102-6.80 × 108). Seven patients suffered from diabetes, 6 from hypertension, one from both diabetes and hypertension, and 2 from rheumatoid arthritis. Clinically, 9 patients exhibited cirrhosis (Child A). Twenty-two patients underwent liver biopsy of which 11 patients showed fibrosis at stage 3 or 4. Cirrhosis and advanced fibrosis were more frequent in relapsers compared to non-responders in this cohort (12/20 vs. 4/24, P = 0.005). The baseline characteristics of study patients are given in Table 1. 

**Table 1.** Baseline Characteristics of Study Patients

Characteristics	Data
**Gender (Male/Female)**	25/19
**Age, y **	
Mean ± S D	43.6 ± 9.4
Median (range)	42.5 (25-70)
**Body mass index, Mean ± SD**	26.8 ± 5.4
**Previous treatment, No.**	
Non-responders	24
Relapsers	20
**Baseline HCV RNA, IU/L**	
Mean ± SD	1.94 × 107 ± 1.08 × 108
Median (range)	7.20 × 105 (6.47 × 102-6.80 × 108)
**Hb%, g/dL, Mean ± SD **	13.0 ± 1.8
**Total leukocyte count (× 10** ^**3**^ **/mcL), Mean ± SD**	6.4 ± 2.1
**Platelets (× 10** ^**3**^ **/mcL), Mean ± SD**	223.7 ± 92.6
**Bilirubin, mg/dL, Mean ± SD**	0.66 ± 0.30
**Alanine aminotransferase, IU/L, Mean ± SD**	67 ± 58
**Gamma glutamyltransferase, IU/L, Mean ± SD**	64 ± 48
**Alkaline phosphatase, IU/L, Mean ± SD**	141 ± 82
**Albumin, g/dL, Mean ± SD**	4.01 ± 0.45
**International normalization ratio (INR), Mean ± SD**	1.14 ± 0.16
**Creatinine, Mean ± SD**	0.7 ± 0.2
**Fasting blood sugar, mg/dL, Mean ± SD**	98 ± 23
**Grade of inflammation (n = 22), No.**	
Mild	3
Moderate	11
Severe	8
**Stage of disease (n = 22)**	
F0	1
F1	3
F2	7
F3	10
F4	1
Steatosis	5

During the first 12 weeks of treatment, two patients developed hepatic encephalopathy and were taken off the study. One patient stopped treatment due to fever and myalgia, and three patients failed to respond. In one patient, HCV RNA was detectable but there was a 2 log reduction. So, early virological response (EVR) was achieved in 37 (84.1%) patients: 22 non-responders and 15 relapsers to previous treatment. At 24 weeks, five patients showed breakthrough when they were on an alternate day regimen. Moreover, one patient with a partial response at week-12 failed to clear the virus. So, at this point, 12 patients were off the study. The rest of 32 patients completed the 48-week treatment (Figure 1). Two more patients were lost to follow-up after completion of treatment leaving behind 30 patients. However, excluding three patients who were dropped out, 41 patients complied with the protocol, so eligible for per-protocol evaluation for end of treatment response (ETR). Two more patients violated the protocol by not-returning for evaluation of SVR, leaving behind 39 patients for per-protocol analysis of SVR. 

According to intention to treat analysis, end of treatment virological response (ETR) was achieved in 19 out of 44 patients (43.2%) which was sustained (SVR) in 12 out of 44 cases (27.3%). With per-protocol analysis, these figures were 19 out of 41 (46.3%) and 12 out of 39 patients (30.8%), respectively. The patients who showed SVR also exhibited normalization of ALT. Comparing responders with non-responders to CIFN plus RBV therapy, only absence of cirrhosis or advanced fibrosis was statistically significant among other factors. (P = 0.032) (Table 2). None of diabetic patients (P = 0.084) but just one patient with hypertension (P = 0.653) achieved SVR. However, the presence of co-morbidity was not statistically significant. 

**Table 2.** Possible Predictors of Sustained Virological Response (SVR)

	SVR, n = 12	No SVR, n = 32	P value
**Gender (Male/Female)**	6/6	19/13	0.567
**Age, mean ± SD, y**	42.6 ± 7.9	44.0 ± 9.9	0.673
**Previous treatment, Non-responders/ Relapsers**	8/4	16/16	0.498
**Body mass index, mean ± SD, kg/m** ^**2**^	24.3 ± 2.9	27.6.0 ± 5.8	0.197
**Co-morbids**	2	14	0.160
**Fasting blood sugar, mean ± SD, (mg/dL)**	87.4 ± 7.5	104.2 ± 27.2	0.086
**Baseline HCV RNA, mean ± SD, IU/L**	2.30 × 106 ± 3.48 × 106	2.45 × 107 ± 1.23 × 108	0.597
**Hb %, mean ± SD, g/dL**	13.3 ± 1.5	12.9 ± 1.9	0.503
**Total leukocyte count, mean ± SD, (× 10 ** ^**3**^ **/mcL)**	6.6 ± 2.6	6.3 ± 1.8	0.666
**Platelets, mean ± SD, (× 10 ** ^**3**^ **/mcL)**	257 ± 117	211 ± 79	0.139
**Bilirubin, mean ± SD, mg/dL**	0.53 ± 0.16	0.71 ± 0.33	0.077
**Alanine aminotransferase, mean ± SD, IU/L**	55± 26	71 ± 66	0.416
**Gamma glutamyltransferase, mean ± SD, IU/L**	49 ± 19	70 ± 53	0.082
**Alkaline phosphatase, mean ± SD, IU/L**	161 ± 114	133 ± 66	0.337
**Albumin, mean ± SD, g/dL**	4.1 ± 0.29	3.9 ± 0.49	0.141
**Advanced fibrosis/ clinical cirrhosis**			0.032
Yes	1	15	
No	11	17	
**Early virological response**	12	25	0.163

Common side effects were fever in 40 (91%), myalgia in 22 (50%), anorexia in 15 (34.1%), depression in 6 (13.6%), pallor in 5 (11.4%), and weight loss in 4 (9.1 %) patients. Most adverse events attributed to CIFN were mild to moderate in severity and short-lived. Two patients on daily CIFN therapy developed neutropenia of less than 0.75 × 103/mcL. It was dealt with through giving G-CSF and temporary dose reduction. Seven patients received erythropoietin for anemia. Six patients received selective serotonin reuptake inhibitors (SSRIs) for mild depressive symptoms, two patients developed portosystemic encephalopathy and were dropped out of the treatment.

## 5. Discussion

Significant morbidity and mortality associated with HCV and treatment failures in approximately half of all patients with Peg-IFN plus RBV remain major concerns for health care providers (9). SVR achievement may halt the progression of fibrosis with lower risk of developing HCC, and improves the survival (11). DAAs are undergoing clinical trials, and interferon-free regimens are still not approved. Triple regimen containing boceprevir or telaprevir, in conjunction with Peg-IFN and RBV, is approved only for genotype 1 (31). Henceforth, there is a need to alternative antiviral therapy from the available armamentarium for CHC genotype 3 patients who are non-responders or relapsers to Peg-IFN and RBV therapy to stop viral replication and ultimate hepatic decompensation, and prevent HCC. CIFN may be a treatment option for this group of patients with CHC (3). However, generalizing results of available clinical studies evaluating CIFN for treatment of who with previous failure in Peg-IFN plus RBV regimen can be challenging. This is mainly due to wide variations in study design, dosing regimens, duration of therapy, and heterogeneous patient populations (based on prior response to therapy) involved in these study. Moreover, data regarding efficacy of CIFN in relapsers were limited, and majority of the patients (90-100%) recruited in these studies were affected by HCV genotype 1; a genotype which responds in a different way from genotype 3.

The preferred dose of CIFN and duration of therapy in the setting of treatment has not been well established (3, 15). The approved dose of CIFN varies from country to country; for instance, in the United States, the approved dose is 15 μg three times a week given subcutaneously, whereas in Germany it is 9 µg three times a week (3, 17, 32). To provide more favorable kinetics and subsequent maximal viral suppression, a daily dosing trial was attempted (17, 33, 34). However, in DIRECT trial, a daily dose of 15 µg was found to be associated with discontinuation of CIFN in 21% patients due to various side effects (33). On the other hand, switching daily dosing to thrice a week regimen for those who achieved EVR could improve the tolerability and compliance for CIFN36. Nonetheless, the overall frequencies of adverse effects and dose modification even with the higher doses of CIFN + RBV therapy are comparable to what was reported for Peg-IFN + RBV therapy (3). 

Our patients received CIFN 15 µg/day along with RBV. If the patients achieved EVR, CIFN dose was reduced to 15µg thrice a week for further 36 weeks. The patients with a partial virological response continued to receive CIFN 15µg daily and RBV. The treatment was discontinued in both groups if HCV RNA remained, or became detectable at 24 weeks. Five patients experienced breakthrough when CIFN dose was reduced to thrice a week after initial EVR. Probably, the trice a week dose was not sufficient enough to keep the viral replication suppressed, and this could have been prevented if daily dosing was continued.

Data regarding effectiveness of CIFN among patients who failed to respond to Peg-IFN and RBV were limited and somewhat conflicting. Overall, a SVR rate of 6% has been reported among non-responders to Peg-IFN and RBV in a retrospective data analysis from US Veterans Administration hospitals when treated with CIFN (35). In another group of patients, only 10.7% of cases of non-responders to Peg-IFN plus RBV achieved SVR after 48-weeks treatment with CIFN 15 µg/day with RBV, whereas lowering the dose of CIFN to 9 µg/day reduced SVR merely to 6.9% (33). Leevy CB retrospectively analyzed 137 patients unable to achieve an early virological response with Peg-IFN + RBV, who were treated with CIFN 15 µg /day plus RBV afterwards (36). The dose of CIFN was reduced to 15 µg thrice a week (TIW) for 36 weeks in that study if the patient was HCV RNA negative at week 12. Overall, 37% of patients could achieve SVR (36). In our study, all-inclusive SVR was 27.3% and in non-responders, it was 33.3% (8/24). 

Previous studies have shown that patients who relapse, exhibit partial response, or experience breakthrough are more likely to achieve SVR with re-treatment compared to patients who showed null response (2, 37). A study showed a SVR achievement of 31% in such patients re-treated with CIFN and RBV (35). Our study performed on the relapsers of genotype 3 could not clinch that much, as SVR was achieved only in 4 out of 20 patients (20%). Our non-responder group showed better off with one-third of patients achieving SVR. 

Due to limited funding, our study was not powered to find out the factors predicting SVR to CIFN with RBV therapy in genotype 3 treatment-experienced patients. Though our patients with co-morbidity demonstrated a low response and all patients with diabetes did not achieve SVR, it could not achieve the significance due to small sample size. Baseline viral load did not influence on the outcome. Advanced fibrosis was the only parameter of significance, which adversely affected SVR. . Just one patient out of 16 patients with F3 or F4 fibrosis or clinical cirrhosis could achieve SVR. It was previously shown that genotype 3 no longer remains a privileged genotype to respond to treatment when fibrosis advances (5, 38). The reason why the non-responders compared to relapsers achieved higher SVR in our study might be due to high proportion of patients with advanced fibrosis and cirrhosis in the latter group.

Currently, available DAA-based regimens for the treatment-experienced patients are approved only for HCV genotype 1. The strength of this study is that we have tried to explore an alternative approach for the retreatment of HCV patients infected with genotype 3. The data regarding use of CIFN and RBV therapy in such patients were scanty. The major weakness of this study appears to be small size of the sample.

In conclusion, about a quarter of HCV genotype 3 patients previously treated with Peg-IFN and RBV benefited from re-treatment with CIFN and RBV. However, the degree of fibrosis influenced the outcome of re-treatment.
